# Implementing a Smartphone and Wearable-Based Stress Management Intervention in Women With Coronary Artery Spasms to Reduce Cardiac Symptoms: Multicenter, Single-Arm, 1-Way Crossover Study

**DOI:** 10.2196/76146

**Published:** 2026-07-28

**Authors:** Peter David Faasse, Annemiek de Vos, Joan G Meeder, Arnoud W J Van ’t Hof, Patty JC Winkler, Jos W M G Widdershoven, Valeria Paradies, Eveline Wouters, Gerard Schouten, Angela H E M Maas, Suzette E Elias-Smale

**Affiliations:** 1 Department of Cardiology Radboud University Medical Center Nijmegen The Netherlands; 2 Department of Cardiology Catherina ziekenhuis Eindhoven The Netherlands; 3 Department of Cardiology VieCuri Medisch Centrum Venlo The Netherlands; 4 Department of Cardiology Maastricht University Medical Centre Maastricht The Netherlands; 5 Department of Cardiology Zuyderland Medisch Centrum Heerlen The Netherlands; 6 Department of Cardiology Elisabeth-TweeSteden Ziekenhuis Tilburg The Netherlands; 7 Department of Cardiology Maasstad Ziekenhuis Rotterdam The Netherlands; 8 School of Allied Health Professions Fontys University of Applied Sciences Eindhoven The Netherlands; 9 School of ICT Fontys University of Applied Sciences Eindhoven The Netherlands

**Keywords:** coronary vasospasms, eHealth, heart rate variability, mental stress, social-psychological factors

## Abstract

**Background:**

Mental stress is a well-known trigger of cardiac symptoms in patients with coronary artery spasms. Hence, stress management is recommended along with medical therapy. However, specific programs for patients with coronary spasms are lacking. We collaborated with patients to develop a smartphone-based app as a tailored solution. The app provides biofeedback based on heart rate variability–driven stress level estimations.

**Objective:**

This study aimed to evaluate the effect of a biofeedback-driven smartphone stress management app on cardiac symptoms in women with coronary vasospasms.

**Methods:**

We enrolled 117 women aged 18 to 70 years diagnosed with coronary vasospasms, as confirmed by a gold standard coronary function test. A multicenter, single-sequence, 2-period crossover study was conducted, comprising a 4-week control period followed by a 4-week period using the Wavy intervention app. The intervention comprised breathing-based exercises that were prompted when measured stress levels were too high. The primary outcome was the Seattle Angina Questionnaire Summary Score, and the secondary outcomes included the 36-Item Short Form Health Survey and the Perceived Stress Scale-10 items. Additionally, the user experience and the impact of the breathing exercise on heart rate variability were evaluated.

**Results:**

A total of 102 patients completed the study, yet no significant improvements were observed in the Seattle Angina Questionnaire following the intervention period, with the control group scores at 50.4 (SD 15.4) and the intervention group scores at 50.9 (SD 15.0; *P*=.92). Similarly, no differences were found in the 36-Item Short Form Health Survey and the Perceived Stress Score. However, the 1236 breathing relaxation exercises performed during the study led to a significant improvement in heart rate variability, as indicated by a median root mean square of successive differences increase from 18.59 (IQR 8.38-36.58) milliseconds before the exercises to 34.93 (IQR 24.19-50.64) milliseconds after the exercises (*z*=−13.72; *P*<.001). Furthermore, two-thirds of participants (68/102, 66.7%) indicated that they would use a more refined version of the app in the future.

**Conclusions:**

A 4-week intervention using the Wavy app did not significantly alleviate anginal symptoms in female patients with coronary vasospasms. However, the breathing exercises demonstrated a notable improvement in heart rate variability, suggesting a reduction in stress levels. Patient feedback indicated broad support for the app and its wearable-based functionality, emphasizing the need for substantial refinements. Future development should incorporate patient perspectives to optimize the app as a comprehensive lifestyle management tool.

**Trial Registration:**

ClinicalTrials.gov NCT06171893; https://classic.clinicaltrials.gov/ct2/show/NCT06171893

## Introduction

The global burden of patients experiencing chronic angina pectoris is high, with a prevalence of 1.5% to 1.7% in the United States from 2019 to 2023 [1]. Nearly two-thirds of women with angina referred for coronary angiography exhibit no obstruction of the coronary arteries [2]. As much as 60% to 90% of these patients appear to have underlying coronary vascular dysfunction, encompassing both epicardial and microvascular coronary spasm endotypes. Both endotypes can be diagnosed using the gold standard coronary function test (CFT) and are collectively classified as coronary artery spasms (CASs) [3-5]. CAS generally affects middle-aged women and typically results in chronic and severe angina, sometimes accompanied by exertional dyspnea, significantly impairing quality of life [6]. Moreover, patients with CAS face an elevated risk of major cardiovascular events [7]. This condition results in work limitations and increased health care use and associated costs [8].

Emotional stress is a well-known trigger for CASs and subsequent angina in this specific patient group [9,10]. Hence, stress reduction is one of the cornerstones in managing CAS, as indicated in the European Association of Percutaneous Cardiovascular Interventions consensus document on CAS [11]. However, specific stress reduction programs tailored to patients with CAS are still lacking.

To address this gap and provide a patient-centered stress management solution, the Wavy Assistant (Wavy) smartphone app was developed. Wavy was designed in 3 iterative phases, in which developers updated the app version according to patient-based feedback. The feedback was derived from 3 small 4-week pilot rounds involving 10 to 20 patients each, and in total, 48 patients participated in these pilot rounds. These patients were also eligible for the study described in this paper. The prototype app integrates breathing-based exercises triggered by elevated stress levels, as detected through a real-time biofeedback system connected to the Garmin Vivosmart 4 [12,13]. This system uses heart rate variability (HRV) as a proxy measure for stress. This study selected HRV as a measure of stress due to its well-established role as an indicator of parasympathetic activity and its inverse relationship with mental stress. Both acute and chronic stress have been consistently associated with decreased HRV, reflecting a shift in autonomic balance toward sympathetic dominance. In contrast, a higher HRV reflects greater parasympathetic (vagal) activity and greater adaptability to stress. Numerous studies have demonstrated that HRV reliably decreases under stressful conditions, underscoring its validity as a physiological proxy for stress [14]. Moreover, higher HRV values have been linked to a reduced risk of cardiovascular disease [15].

Aiming for a proof of concept, this study evaluated the impact of the prototype Wavy app on cardiac symptoms, quality of life, and stress levels to determine its potential as an effective stress management tool.

## Methods

### Patient Population

Patients with coronary vascular dysfunction were screened for enrollment in 6 Dutch hospitals: Radboud University Medical Center Nijmegen, Maastricht University Medical Center, Zuyderland Hospital Heerlen, St Elisabeth Hospital Tilburg, Catharina Hospital Eindhoven, VieCuri Hospital Venlo, and Maasstad Hospital Rotterdam.

Adult women were eligible if diagnosed with CAS, defined as coronary epicardial vasospasm or microvascular spasm determined by CFT with acetylcholine according to the criteria of the Coronary Vasomotor Disorders International Study Group [5,16].

The upper age limit for inclusion was 70 years to enhance digital literacy. Further exclusion criteria included obstructive coronary artery disease (CAD), defined as ≥70% coronary artery stenosis, fractional flow reserve <0.80, or both; and practical inability to participate in the study, for example, due to a language barrier.

### Study Design

The study was designed as a single-sequence, 2-period, crossover study, allowing a within-patient comparison between control and intervention. After signing the informed consent form, participants had 1 habituation week to get used to the Garmin Vivosmart 4 wearable.

During this week, the researchers had a one-on-one digital or in-person meeting with the patient, setting up the connection and addressing potential questions and issues. The patients were instructed to wear the wearable during the daytime; nighttime use was optional. The 4-week control period was initiated when Bluetooth successfully connected the wearable.

After the control period, the consecutive 4-week intervention period commenced, in which the Wavy app was updated to the full access version. The researchers guided patients in installing the Wavy app, and patients were personally informed about the available features mentioned in the Wavy Assistant App section below. Considering that the study used a prototype version of the app, technical support was a priority and was readily accessible throughout the study period. Incoming data were closely monitored to promptly identify and address any technical issues. If no data were received for 2 consecutive days, researchers proactively initiated technical support. This support was provided through one-on-one virtual meetings, which included assisted screen sharing to resolve issues efficiently.

### Wavy Assistant App

#### Intervention App

**Figure 1:**
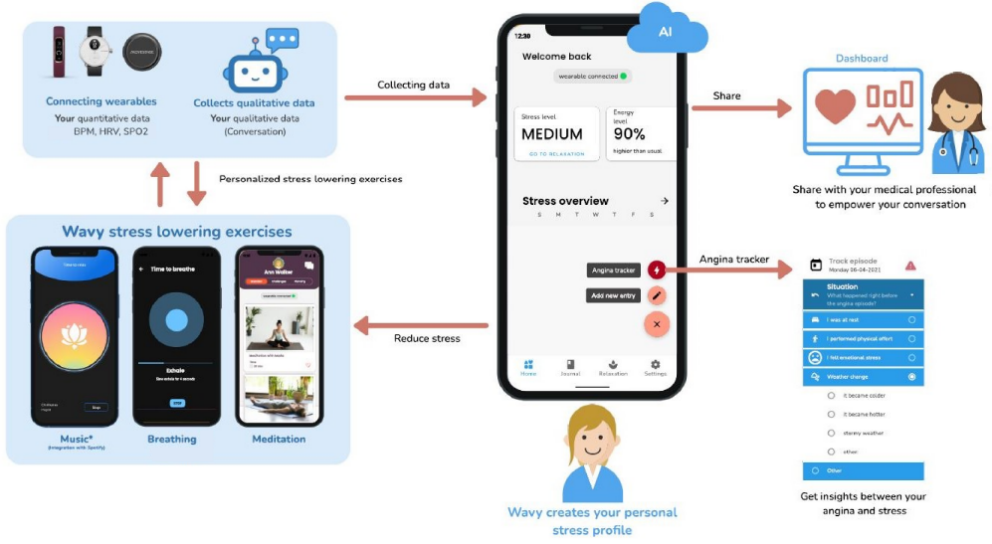
Visualizing Wavy: the app is installed on a smartphone (with either an Android or iOS operating system). This app retrieves data from the Bluetooth-connected wearable (in this case, the Garmin Vivosmart 4). On the basis of the raw beat-to-beat intervals data from the wearable, stress-related features are computed (particularly the root mean square of successive differences). After a 1-week baseline, personalized thresholds for stress are determined, which are updated continuously based on a rolling average. In case of exceeding the thresholds, a notification is sent, recommending a box breathing exercise. Furthermore, the app can track angina and assist with physician appointments in the future. HRV: heart rate variability.

The prototype app version (Figure 1) integrates data from a wearable device to continuously monitor stress levels and provide real-time biofeedback, leading to the suggestion of breathing exercises if elevated stress levels are detected. Future features of different relaxation exercises and data sharing were not yet included. The prototype uses a machine learning algorithm based on wearable data, specifically the heart rate and HRV, from the Garmin Vivosmart 4. This smartwatch uses photoplethysmography to measure beat-to-beat intervals and supplies the data to Wavy in real-time via Bluetooth. The wearable was also used by Carreiro et al [17] for stress-related research. Apart from supporting HRV measurements, the wearable is small and has a 7-day battery life, making it suitable for the current research phase. Upcoming Wavy iterations aim to be device agnostic.

**Figure 2:**
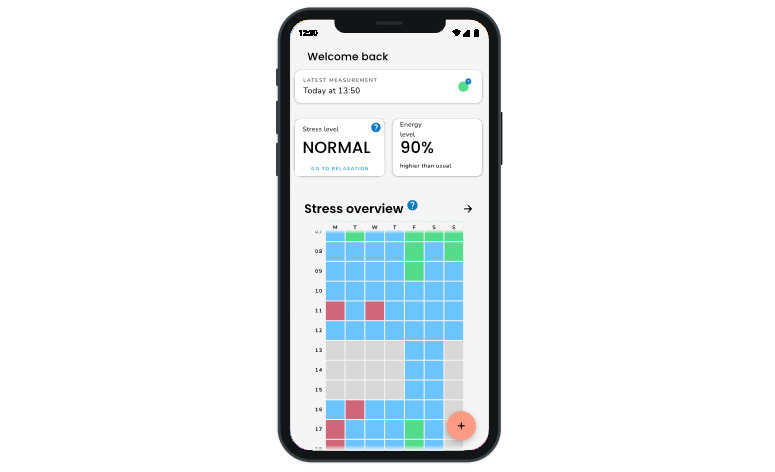
Stress overview. The home page is displayed, showing the connection status, the latest stress level, and an hour-by-hour stress overview for the past week.

**Figure 3:**
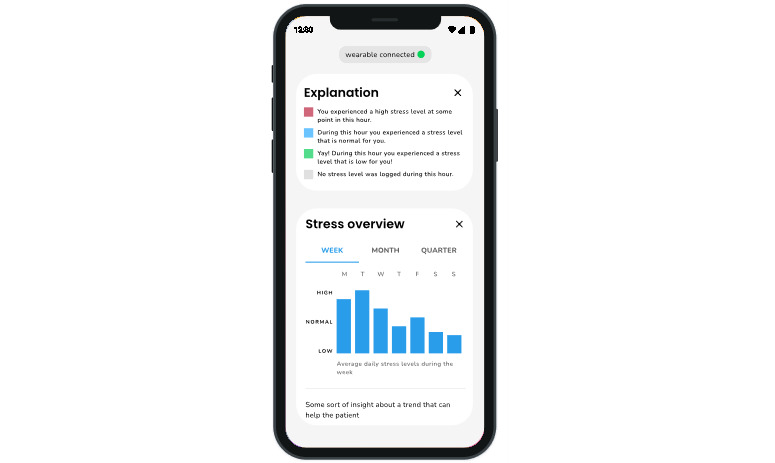
Explanation of the overview provided for the patients, along with a longer-term stress overview.

The HRV metric served as the proxy for stress and was calculated as the root mean square of successive differences (RMSSD) in milliseconds [14,18]. This metric was computed from beat-to-beat interval, as measured in the background with ultrashort frames of 30 seconds every 15 minutes and continuously during the breathing exercise. These ultrashort time frames capture the HRV RMSSD values well, as concluded by the validation study of Munoz et al [19]. On the basis of the RMSSD values, Wavy displayed a color-coded overview of their stress patterns per hour for the past week (Figures 2 and 3).

If the app estimated stress levels higher than 1.5 times the patient’s SD, a notification was sent that offered a brief stress-reducing exercise. The exercise entailed a visually guided box breathing exercise with 4 seconds in, hold, out, and hold stages [20]. Patients were told that shorter intervals were acceptable if they had shortness of breath.

#### Blank Control App

To evaluate the effect of the Wavy app, Wavy needed to be compared to a control version. The “Blank” app solely displayed the connection status of the wearable device without providing any additional feedback or intervention. Similar to the Wavy app, the Blank version allowed symptom registration for this trial.

### Statistical Analysis

#### Outcome Measures

The main objective was to assess the effect of Wavy on anginal burden. To this end, the primary outcome was the difference in the average Seattle Angina Questionnaire Summary Score (SAQ-SS) [21], measured after 4 weeks of intervention with the Wavy app vs the control version. For reference, a score of 0 to 24 indicates poor, 25 to 49 fair, 50 to 74 good, and 75 to 100 excellent health status. As secondary outcomes, we examined differences in quality of life using the 36-Item Short Form Health Survey (SF-36) [22] and the Perceived Stress Score-10 items [23], which were administered at baseline and after the 4-week control and 4-week intervention periods. The validated questionnaires assessed experiences over the past month, allowing for a clear comparison between the 1-month control and intervention periods.

Furthermore, we examined the effect of the breathing exercises on estimated stress levels as measured with HRV, expecting stress-reducing results. Thus, HRV values before, during, and after exercises were analyzed.

Given that the app was still under development, we monitored app stability based on incoming user data and participants’ feedback or support requests.

#### Sample Size Calculation

A 2-sided sample size calculation was performed with β of .8 and α of .05. The calculation was based on the primary outcome of the SAQ-SS; an alteration of 5 points is generally regarded as clinically relevant. The reference SD was estimated to be 15 based on patients with stable CAD, as reported by Chan et al [21] and others [24,25]. This calculation indicated a required sample size of 97 patients. To account for potential technological challenges, we added 20% to correct for the potential dropout rate; thus, 117 patients were to be included.

#### Questionnaires

Descriptive statistics were reported as mean (SD) or median (IQR), depending on the normality of the data distribution. Questionnaire-based outcomes were analyzed with a paired *t* test between the control and intervention months.

Statistical analysis of questionnaire-based data was performed using SPSS Statistics (version 22.0; IBM Corp). All statistical tests were 2-tailed, and *P*<.05 was considered statistically significant.

#### Wearable Data Analysis

In analyzing this semicontinuous HRV data, we focused on the relaxation exercises in the intervention month. To this end, the last available HRV measurements were taken before the exercise, during the exercise itself, and the first postexercise measurement. Exercises with a duration shorter than 1 minute were filtered out. Considering the measurement interval was 15 minutes, the before and after measurements were between 0 and 15 minutes apart from each exercise. The effect of the exercise on stress was determined by a Wilcoxon test of the HRV measurements before, during, and after the exercise.

A skewed distribution was expected as RMSSD has a lower limit of 0. Thus, results are displayed as the median (IQR).

According to the hypothesis, the HRV should increase during the exercise, suggesting decreased stress, and continue to be elevated, albeit to a lesser extent, in the first postexercise measurement.

HRV analysis was performed using Python (version 3.1; Python Software Foundation) with the Pandas software library.

#### Data Management

All data were pseudoanonymized, and the encryption key linking identifiers was stored on a secure hard drive at Radboudumc. Questionnaire data were collected through the Castor Electronic Data Capture System and stored on a secure Radboudumc hard drive for 15 years.

The coordinating researcher provided pseudoanonymized log-in codes for patients to access the app for HRV data. The pseudoanonymized code was used in the data collection to ensure that Wavy did not gain access to personal information. The HRV data were stored in the European OVH cloud, which is certified for storing medical data.

### Ethical Considerations

All participants provided written informed consent before participation. The study procedures comply with the ethical standards of the Declaration of Helsinki (2013). Moreover, the central commission of human related research region Arnhem-Nijmegen reported no objections to conducting the study, and local ethics approval was acquired for each center (NL77493.091.21). Pseudoanonymized data safeguarded patients privacy. Patients received no financial compensation for participation. Reporting is done according to the digital health implementation iCHECK-DH [26].

## Results

### Patient Inclusion

**Figure 4:**
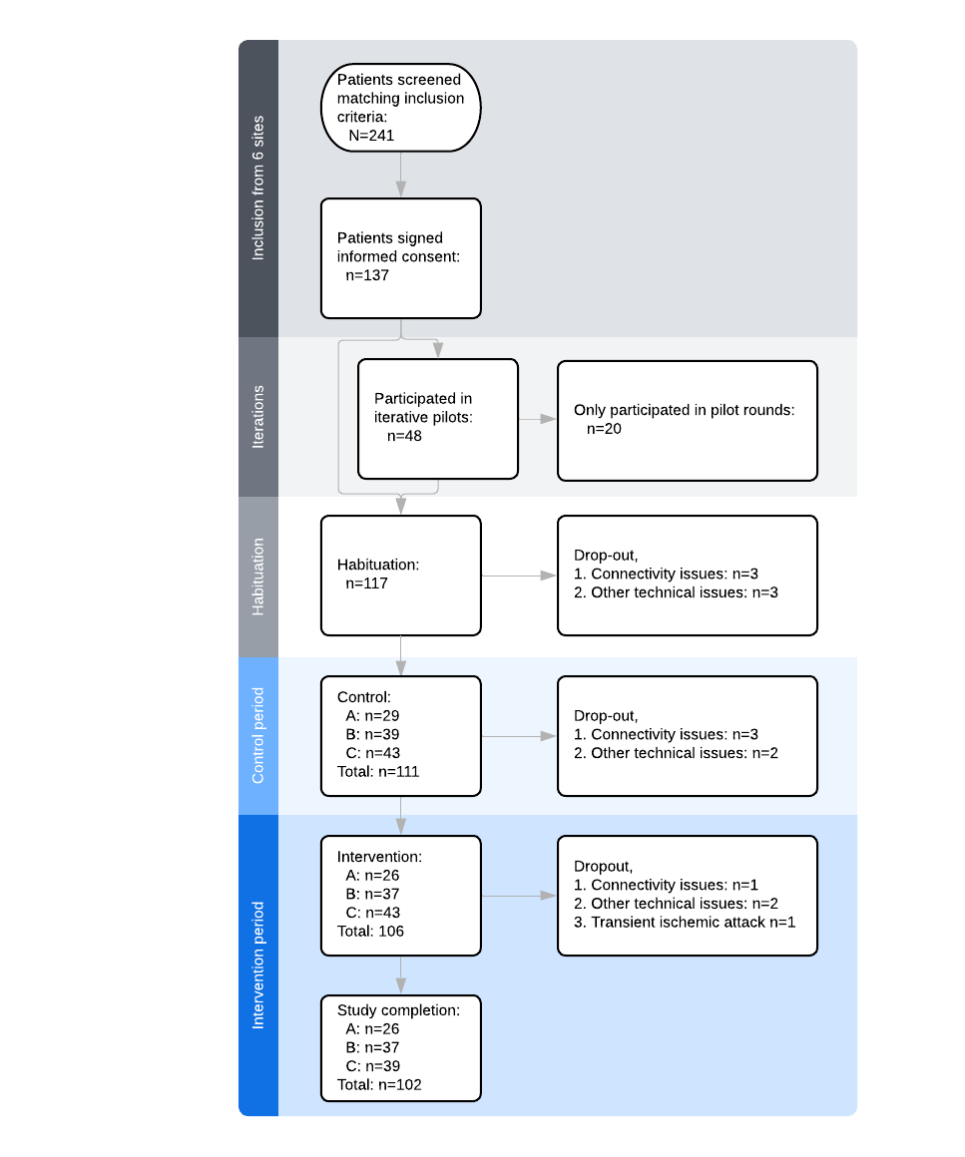
Study flowchart, inclusion and development phases in grey tints and study phases in blue tints. In total, 241 patients were screened, of whom 137 provided written informed consent. In total, 48 patients participated in pilot trials, of whom 28 continued to the final trial. Consequently, 117 patients started the study. Unfortunately, 15 dropped out due to technical issues. The rest were split into different starting waves (A-C) and completed the trial.

We screened 241 women with CAS, who were diagnosed by the CFT with acetylcholine. Of those, 137 (56.8%) provided informed consent. A total of 48 (19.9%) patients participated in the smaller pilot iteration rounds, aiming to develop and improve the Wavy app further, of which 28 (58.3%) continued to this study. The resulting 117 (48.5%) patients were included in the main trial described in this paper. Technological barriers and other issues with the app prohibited proper app use for 15 (12.8%) patients, leaving 102 (87.2%) patients to finish the trial (Figure 4). The trial enrollment was split into 3 periods, with group A starting in August 2022, group B in September 2022, and group C in November 2022.

### Evaluation Outcomes

Baseline characteristics are described in Table 1. The mean age was 58 (SD 7.3) years. From the 102 patients, the CFT diagnosed 53 (51.9%) patients with epicardial spasm and 49 (48%) patients with microvascular spasm as their endotype of CAS according to the Coronary Vasomotor Disorders International Study Group guidelines. The mean study duration was 59 (SD 9.2) days. The average number of days with a successful HRV measurement was 49 (SD 10.6); on the remaining days, no measurement was registered. On days with HRV registration, 61% of the maximum possible measurements were successfully recorded. In total, 1236 breathing exercises were performed with an average duration of 1 minute and 43 second (SD 43 seconds).

**Table 1:** Baseline characteristics (N=102).

Characteristics		Values
Age (years), mean (SD)		58 (7.32)
Sex (female), n (%)		102 (100)
**Etiology, n (%)**		
	Epicardial	53 (52)
	Microvascular	49 (48)
**Baseline questionnaires, mean (SD)**		
	SF-36^a^ summary score	52.9 (14.0)
	SF-36 mental component	55.6 (15.5)
	SF-36 physical component	51.7 (15.4)
	Seattle Angina Questionnaire Summary Score	49.6 (16.8)
	Perceived Stress Score-10 items	19.7 (6.9)

^a^SF-36: 36-Item Short Form Health Survey.

**Figure 5:**
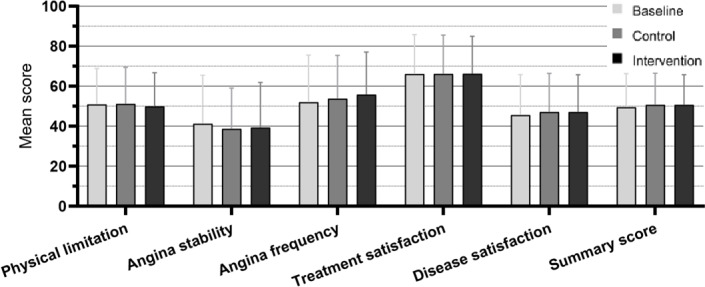
Seattle Angina Questionnaire Summary Score, components, and summary scores.

**Figure 6:**
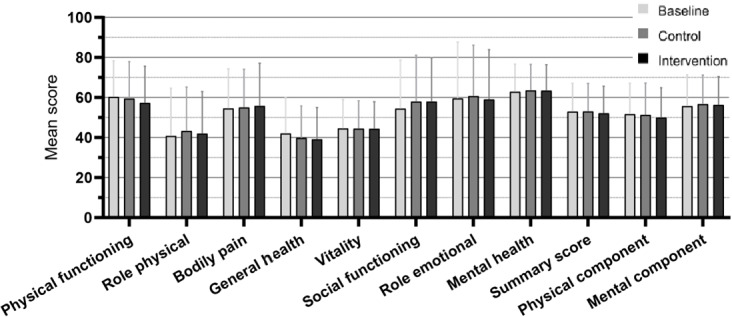
36-Item Short Form Health Survey: components and summary score.

**Figure 7:**
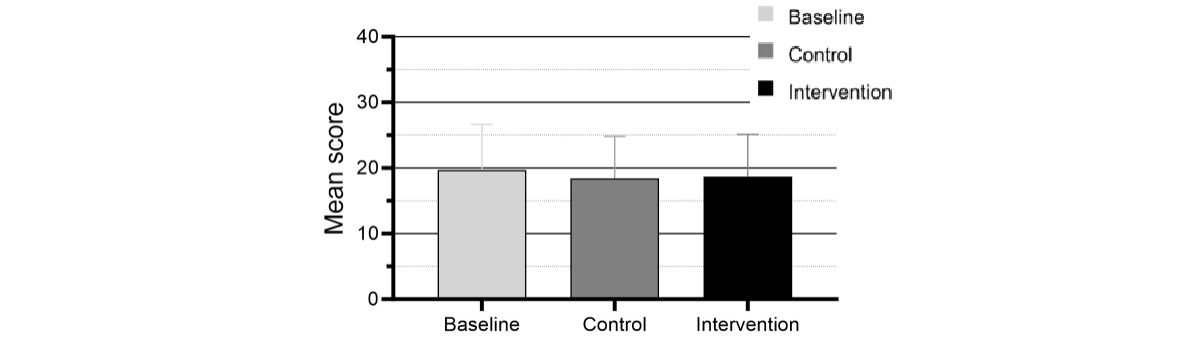
Perceived Stress Score.

The primary outcome, average SAQ-SS, following the control and intervention phases was 50.4 (SD 15.4) and 50.9 (SD 15.0), respectively. These values indicated a fair to good quality of life in both groups, and comparison exhibited no statistically significant difference (*P*=.59; Figure 5). The mean SF-36 summary scores and individual components did not differ significantly (*P*=.92; Figure 6). Furthermore, the mean perceived stress score also showed no statistical difference (Figure 7).

**Figure 8:**
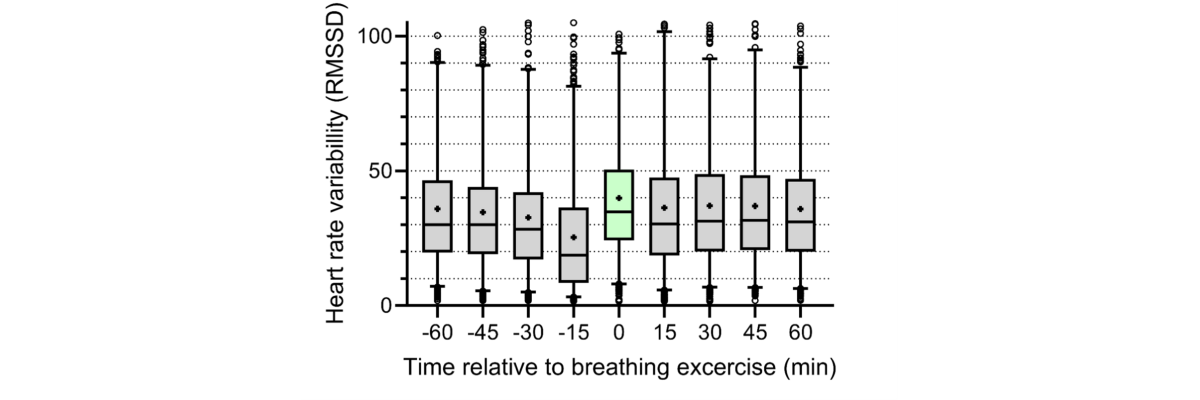
Breathing exercises on heart rate variability. This displays the relative time points to the breathing exercises. RMSSD: root mean square of successive differences.

However, HRV analysis showed a significant increase in RMSSD, indicating a relaxing effect during and after breathing exercises, compared to the measurement 15 minutes before, with a median (IQR) RMSSD of 18.59 (8.38-36.58) milliseconds before and 34.93 (24.19-50.64) milliseconds during the relaxation exercise (Z=–13.72; *P*<.001). Compared to the previous measurement, this effect is attenuated at 15 minutes after the exercise with a normalized median of 30.72 (IQR 18.97-47.76) milliseconds (Z=–11.75; *P*<.001). A visual overview is displayed in Figure 8.

Regarding the user experience, the Wavy app, which was still in development, experienced technical instability. With measurements taken every 15 minutes, a total of 96 time points could be collected each day, and 61.5% (59/96) of the possible data points were retrieved. Technical issues were prevalent, with most patients reporting technical issues, including difficulties logging in, connecting the wearable to the phone, and crashing. These issues resulted in increased technical support from the researchers. Nevertheless, 68 (66.7%) of 102 patients reported that they would use such an app in the future, provided it were a stable version.

## Discussion

### Principal Results

The study was conducted to assess the impact and potential of the prototype Wavy app that strives to aid in lifestyle management for patients with CAS. As per the inclusion target, 102 (87.2%) of 117 patients completed the study. The primary outcome, the SAQ-SS, and secondary measures (SF-36 and Perceived Stress Score-10 items) did not exhibit significant improvements after a 4-week intervention period. However, the study was hampered by technical difficulties related to the app being in its development phase.

Notably, the results of the HRV analysis indicated increased HRV levels during the breathing exercise, with normalized values after the breathing exercise, thus showing a significant positive effect of breathing exercises on stress levels.

### Comparable Studies

In reviewing the literature, no comparable studies of HRV biofeedback–based stress management on cardiac symptoms and quality of life were found for this specific patient group. However, various studies investigated the effect of HRV biofeedback–based stress management on mental stress and anxiety levels. Goessl et al [27] conducted a meta-analysis of 24 studies investigating the stress-reducing effects of HRV biofeedback. The analysis spanned 10 clinical and 14 community settings, with the majority of research (n=18) originating from the United States and the European Union [28,29]. In total, 13 studies that were mentioned by Goessl et al [27] included a comparison group, constituting standard care, sham, or other means of stress reduction. In some studies, the HRV biofeedback training could be performed at home with a device, while others included a personal trainer; the frequency of HRVB sessions varied between 1 and 50. When comparing the HRV-based stress management to the comparison condition, the biofeedback resulted in an effective reduction of anxiety and stress [27]. While Wavy did not achieve the desired effectiveness in improving perceived stress scores, we found a significant HRV improvement, indicating stress reduction.

In contrast to our intervention, which involves providing breathing exercises upon the detection of increased stress to enhance HRV and subsequently reduce stress, Held et al [30] explored the reduction in HRV induced by exposure to a stressful working condition in 26 participants with clinical anxiety and 14 control participants. Notably, the magnitude of HRV changes observed in both groups closely resembled the inverse HRV results presented in our paper. The decline in HRV during a cognitive stressful task in both healthy individuals and individuals with anxiety aligns with the HRV increase observed during the breathing exercises for these patients. Consistent with the findings of Held et al [30], the magnitude identified in our HRV analysis indicates a relevant stress reduction.

In comparison to other remote HRV biofeedback studies, compliance was decent, with 1236 breathing exercises performed (approximately 3 per week per patient). In contrast, a similar study by Hirten et al [30] failed to demonstrate the feasibility of remote monitoring due to poor compliance. Remote, wearable-based studies face significant challenges in acquiring a representative sample of available data points. For instance, Galarnyk et al [31] reported that only 5.6% of all recorded beat-to-beat interval data were usable, with 43% of participants never activating their device. In this study, despite similar technical challenges, 61.5% (59/96) of the total achievable data points—each representing approximately 30 seconds of recording every 15 minutes—were successfully collected. This outcome underscores the importance of active data monitoring and proactive technical support in maximizing data retention. Additionally, it highlights the role of an initiative-taking patient population, which likely contributed to the relatively high data capture rate observed in this study.

### Strengths and Limitations

Although stress management is included in the current guidelines for the management of coronary vasospasms, our study is the first to evaluate a stress reduction program in women with proven CASs. The fact that 66.7% (68/102) of patients expressed willingness to engage in further app use after stabilization underscores the willingness of the patients to apply a home-based, digital form of stress management.

This study design enabled each patient to serve as their own control, reducing the influence of confounding variables. Although factors such as disease progression or medication changes could have introduced some variability, the 10-week study period makes the impact of these factors unlikely to be substantial.

As the app was still in the development phase, we encountered more technical issues than anticipated. While nearly all challenges were addressed through active data monitoring and personalized support, including screen sharing sessions, these recurring efforts placed an additional burden on participants and likely impacted their overall user experience. Consequently, it is difficult to draw definitive conclusions about the program’s effectiveness in alleviating symptoms. Stress is one of the potential triggers of angina; therefore, a full disease management app should extent their functionalities to address other lifestyle factors, for example, blood pressure management.

Despite these technical challenges, HRV analysis demonstrated a positive effect of the breathing exercises on stress reduction. Additionally, unlike many stress management interventions reported in the literature, which often span longer durations, our study used a relatively short intervention period. Considering evidence suggesting that extended intervention durations may yield greater improvements in self-reported stress and anxiety, the limited time frame of this study may have been a key limitation [27].

Because the SAQ-SS was developed primarily for obstructive CAD, its applicability to nonobstructive coronary disease is debated, and this may have been a potential limitation [32]. However, other studies support this psychometric for a more widespread population, including nonobstructive CAD [25,33]. A more sensitive CAS-specific outcome measure may yield more insightful results in future research; however, this has yet to be developed.

### Conclusions

In conclusion, the current digital stress reduction prototype, Wavy app, did not affect perceived anginal burden in female patients with coronary vasospasms as measured by various questionnaires. However, considering the technical limitations of the device in its development stage, it is too early to dismiss the potential of this app to provide biofeedback to complement existing treatment strategies. The significant improvement in HRV during and after the breathing exercise, indicating the potential stress-reducing effect of the stress management app, is encouraging. For a complete disease management tool, development should extend beyond stress as its sole focus, as stress is just one of the possible symptom-provoking factors.

Future research is warranted with a technically stable device and longer follow-up duration to establish the true effect of stress reduction with breathing exercises on stress symptoms in the growing CAS population.

## Data Availability

The data derived from this study are not publicly available due to privacy and ethical restrictions but can be obtained from the corresponding author upon reasonable request.

## Abbreviations

CAD: coronary artery disease
CAS: coronary artery spasm
CFT: coronary function test
HRV: heart rate variability
RMSSD: root mean square of successive differences
SAQ-SS: Seattle Angina Questionnaire Summary Score
SF-36: 36-Item Short Form Health Survey
